# Carbon Catabolite Repression in Filamentous Fungi Is Regulated by Phosphorylation of the Transcription Factor CreA

**DOI:** 10.1128/mBio.03146-20

**Published:** 2021-01-05

**Authors:** Leandro José de Assis, Lilian Pereira Silva, Ozgur Bayram, Paul Dowling, Olaf Kniemeyer, Thomas Krüger, Axel A. Brakhage, Yingying Chen, Liguo Dong, Kaeling Tan, Koon Ho Wong, Laure N. A. Ries, Gustavo H. Goldman

**Affiliations:** a University of Exeter, MRC Centre for Medical Mycology, Exeter, United Kingdom; b Faculdade de Ciências Farmacêuticas de Ribeirão Preto, Universidade de São Paulo, Ribeirao Preto, Brazil; c Biology Department, Maynooth University, Maynooth, Kildare, Ireland; d Leibniz Institute for Natural Product Research and Infection Biology, Department of Molecular and Applied Microbiology, Institute of Microbiology, Friedrich Schiller University, Jena, Germany; e Faculty of Health Science, University of Macau, Macau, China; f Institute for Advanced Study, Technical University of Munich, Garching, Germany; Karlsruhe Institute of Technology (KIT)

**Keywords:** CreA, carbon catabolite repression, xylanase, biofuels, *Aspergillus nidulans*

## Abstract

Filamentous fungi of the genus *Aspergillus* are of particular interest for biotechnological applications due to their natural capacity to secrete carbohydrate-active enzymes (CAZy) that target plant biomass. The presence of easily metabolizable sugars such as glucose, whose concentrations increase during plant biomass hydrolysis, results in the repression of CAZy-encoding genes in a process known as carbon catabolite repression (CCR), which is undesired for the purpose of large-scale enzyme production. To date, the C_2_H_2_ transcription factor CreA has been described as the major CC repressor in *Aspergillus* spp., although little is known about the role of posttranslational modifications in this process. In this work, phosphorylation sites were identified by mass spectrometry on Aspergillus nidulans CreA, and subsequently, the previously identified but uncharacterized site S262, the characterized site S319, and the newly identified sites S268 and T308 were chosen to be mutated to nonphosphorylatable residues before their effect on CCR was investigated. Sites S262, S268, and T308 are important for CreA protein accumulation and cellular localization, DNA binding, and repression of enzyme activities. In agreement with a previous study, site S319 was not important for several here-tested phenotypes but is key for CreA degradation and induction of enzyme activities. All sites were shown to be important for glycogen and trehalose metabolism. This study highlights the importance of CreA phosphorylation sites for the regulation of CCR. These sites are interesting targets for biotechnological strain engineering without the need to delete essential genes, which could result in undesired side effects.

## INTRODUCTION

In recent years, the conversion of plant biomass waste to biofuels, in a process called 1st- (1G) and 2nd-generation (2G) biofuel production, has gained significant interest as an environmentally friendly and naturally abundant alternative renewable energy source. Saprobic microorganisms, such as filamentous fungi of the genus *Aspergillus*, are of particular interest in this process as they secrete a vast array of plant biomass-hydrolyzing enzymes. Plant biomass is composed of complex carbohydrates (cellulose and hemicelluloses), and enzymatic hydrolysis of these complex carbohydrates by the simultaneous action of several plant biomass-degrading enzymes results in the release of monosaccharides (e.g., glucose and xylose) that are used in fermentation processes to generate renewable biofuels. The genes that encode these enzymes are under tight transcriptional control (1, 2) and are repressed in the presence of easy metabolizable sugars such as glucose, whose concentrations increase during plant biomass hydrolysis.

Glucose-mediated repression of these enzyme-encoding genes is known as carbon catabolite repression (CCR) (1, 3, 4) and is undesired for the purpose of large-scale enzyme production (2, 5). To date, the C_2_H_2_ transcription factor (TF) CreA has been described as the major regulator of CCR in *Aspergillus* spp. (1, 3, 6), although it has become evident that its role goes beyond that of solely a CC repressor, with suggested roles in amino acid metabolism, establishment and progression of infection, enzyme production, carbohydrate storage, production of secondary metabolites (SM), and chromatin remodeling (7–13). In Aspergillus nidulans, truncation of *creA* resulted in strain *creA^d30^*, which had significantly reduced intracellular concentrations of the carbohydrate storage compound trehalose (9). This effect on cellular trehalose concentrations was also observed for the Aspergillus fumigatus Δ*creA* strain (14). In addition, glycogen metabolism in fungi is regulated by CreA homologues, with the deletion of nonessential *creA* causing a hyperaccumulation of glycogen in these fungi (14–16). In A. fumigatus, a preliminary *in silico* analysis found a putative CreA binding site in the 5′ untranslated region (UTR) of the glycogen debranching enzyme-encoding gene *gdbA*, whose expression was significantly reduced in the Δ*creA* strain (14). Furthermore, in A. fumigatus, increased accumulation of intracellular glycogen is controlled by the protein kinase A (PKA) pathway (14), suggesting that PKA is involved in the direct or indirect regulation of CreA.

**FIG 1:**
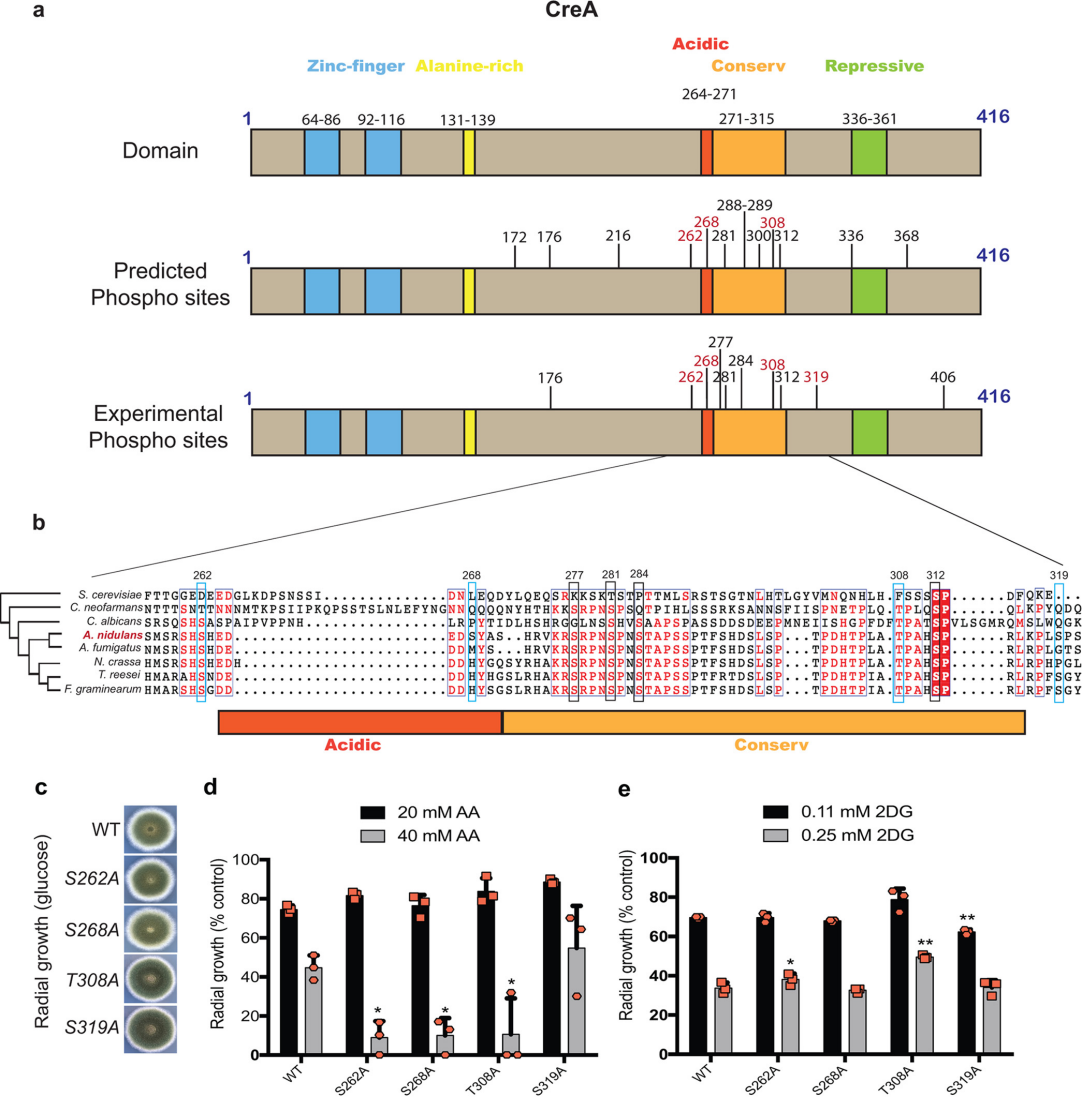
Diagram of CreA protein domains and phosphorylation sites. (a) Schematic diagrams of CreA protein domains. The top shows CreA protein domains and their localization: zinc fingers (blue), alanine rich (yellow), acidic (dark orange), conserved (light orange), and repressive (green). The middle shows CreA phosphorylation sites, as predicted by NetPhos3.1 on the entire length of the protein. The bottom shows CreA phosphorylation sites, as identified by nLC-MS/MS, when the wild-type strain was exposed to 2% (wt/vol) glucose for 30 min. In red are the sites that were experimentally confirmed in this work. (b) Alignment of A. nidulans CreA and homologues of different fungi (yeast and filamentous). The phylogenetic tree shows the closeness between proteins from each fungus; conserved regions are shown in red inside the boxes, and a red box means conserved in all organisms. Highlighted in blue are the phosphorylation sites characterized in this work; highlighted in black are additional phosphosites that were experimentally identified. (c) Strains were grown from 10^5^ spores on glucose minimal medium (MM) for 5 days. Graphs representing radial growth (colony diameter) of strains in glucose (d) or xylose minimal medium (MM) supplemented with increased concentrations of 2DG (2-deoxyglucose) and AA (allyl alcohol) (e). Growth is given as the percentage normalized to the control (without 2DG, AA) condition for each strain. Standard deviations represent the averages from three biological replicates (depicted in orange). *, *P* < 0.05; **, *P* < 0.001 in a two-way ANOVA multiple-comparison test using the WT (wild-type) strain as a reference for each condition.

In A. nidulans, the CreA protein is composed of 416 amino acids, with two N-terminal zinc finger domain regions at amino acids 64 to 86 and 92 to 116 and an N-terminal alanine-rich region composed of amino acids 131 to 139 (Fig. 1a). The C-terminal region of CreA contains the acidic region, located at amino acids 264 to 271, a highly conserved region (called conserved because it is almost identical between A. nidulans, Aspergillus niger, and Trichoderma reesei) at amino acids 271 to 315, and a repressive region composed of amino acids 336 to 361, which has been shown to be essential for repression (Fig. 1a) (2, 13, 17). CreA represses xylanase-encoding genes such as *xlnA*, *xlnB*, and *xlnD* by directly binding to the 5′-SYGGRG-3′ promoter consensus region (18). Furthermore, CreA also regulates *alcR*, the TF that adjusts ethanol metabolism via alcohol dehydrogenase activity (19). Repression of target genes by homologues of CreA has been studied in detail in various fungi (8, 20–22), whereas few studies exist that investigate regulation of CreA itself. Mechanisms of CreA regulation comprise (i) inclusion and exclusion of the nucleus in a carbon source-dependent manner, (ii) protein-protein interactions, and (iii) posttranslational modifications. Correct cellular localization of CreA (1, 3, 23) was not sufficient for repression alone, suggesting that CreA undergoes posttranslational modifications and/or interacts with corepressors such as RcoA and SsnF, which have been shown to be required for full functionality (24–26). A. nidulans CreA is degraded in the presence of the alternative carbon source xylan (CC-derepressing condition), a process that is mediated by the ubiquitylation-dependent Fbx23 F-box complex (24). To date, it remains unclear whether CreA or an interaction partner of this TF is subject to ubiquitylation (13, 24). In addition to A. nidulans CreA interacting with the corepressors RcoA and SsnF, it also physically interacts with glycogen synthase kinase A (GskA) and casein kinase A (CkiA) under CC-derepressing conditions (24). Deletion of *gskA* results in a strain that is severely compromised in growth; however, studies using GskA-green fluorescent protein (GFP) showed that in the presence of glucose, GskA is predominantly cytoplasmic and loses interaction with the CreA complex (24). On the other hand, CkiA is localized throughout the cytoplasm and nucleus (27), and studies using thiamine promoter-induced repression of *ckiA* showed that CkiA is involved in CCR (24).

Interactions with protein kinases is further evidence that CreA is subject to posttranslational modifications such as phosphorylation. CreA was shown to be phosphorylated at S319, with an S319A mutation causing hyperaccumulation of CreA within the cell (28). Additional phosphorylation sites on CreA have also been identified under CCR conditions, including S262, S288, S289, and S319, whereas sites S262 and S288 were identified under CC-derepressing conditions; although, these sites remain uncharacterized (29). In *T. reesei*, mutation of the CRE1 (CreA homologue) serine 241 to alanine (S262 in A. nidulans) resulted in impaired DNA binding and an alleviation in CCR (30). Genes encoding CreA and its homologues are essential depending on the fungal species.

In this work, we identified phosphorylation sites by liquid chromatography-tandem mass spectrometry on A. nidulans CreA under CCR conditions. Subsequently, we further characterized four sites predicted to be phosphorylated by the protein kinases CkiA (S262 and S268), GskA (T308), and Stk22 (indirectly regulated by PkaA-S319) by mutating them to an alanine, thus creating sites that cannot be phosphorylated (hypophosphorylation). We show that these sites are crucial for CCR and CreA cellular localization in the presence of glucose and the hemicellulose xylan as well as for DNA binding, xylanase gene expression, and enzyme activity. Furthermore, these CreA phosphorylation sites are important for glycogen and trehalose metabolism. Finally, this study shows that these point mutations affect CreA protein stability and/or accumulation under different conditions. In summary, this is a comprehensive study that further characterizes the importance of posttranslational modifications on the regulator of CCR CreA in the reference fungus A. nidulans and provides further insights into the complex dynamics that underlie the utilization of carbon sources in filamentous fungi.

## RESULTS

### Identification of phosphorylation sites on CreA.

Previous work has shown that CreA physically interacts with the protein kinases glycogen synthase kinase A (GskA) and casein kinase A (CkiA) under carbon catabolite (CC)-derepressing conditions, suggesting targeted phosphorylation of this TF (24). We therefore decide to first scan for predicted GskA- and CkiA-mediated phosphorylation sites on CreA using NetPhos 3.1 (31, 32). Phosphorylation sites catalyzed by these two protein kinases were predicted to occur at S172, S176, S216, S262, S268, S281, S288, S289, T300, T308, S312, S336, and S368, with the majority of these sites occurring within the CreA acidic and conserved domains (Fig. 1a). To identify CreA posttranslational modifications *in vivo*, liquid chromatography-tandem mass spectrometry (LC-MS/MS) was carried out for the detection of phosphorylation, ubiquitylation, and sumoylation sites when the CreA-GFP strain was grown in minimal medium (MM) supplemented with xylan (CC-derepressing condition) for 24 h and after the addition of 2% glucose (induction of CCR) for 30 min (data available in the PRIDE [Proteomics Identification Database] under accession number PXD018967). Subsequently, immunoprecipitation (IP) of the CreA-GFP fusion protein was carried out. We included ubiquitylation and sumoylation in order to determine whether CreA is subject to posttranslational modifications other than phosphorylation, especially as ubiquitylation has been shown to play a role in CreA protein dynamics (13). We were unable to identify statistically significant CreA posttranslational modifications in the presence of xylan, likely due to CreA being degraded in the cytoplasm under CC-derepressing conditions (13, 24). In the presence of glucose (CCR), ubiquitylation and sumoylation were not identified on CreA. Both posttranslational modifications are related to degradation processes, with ubiquitylation being used to tag proteins for 26S proteasome degradation and sumoylation being targeted by SUMO ubiquitin ligases that can use SUMO moieties to introduce ubiquitylation (33). In agreement with previous studies (13, 24), these results suggest that CreA is not directly targeted for degradation by ubiquitylation and sumoylation processes in the presence of glucose. In contrast, several phosphorylation sites were identified under CCR conditions (see Table S1 available at 10.6084/m9.figshare.13181990). Again, the majority of these sites were identified near to and/or within the acidic and conserved domains (Fig. 1a), both of which are required for CreA-GFP translocation into the cytoplasm under CC-derepressing conditions (13).

To determine whether these phosphorylation sites are also present in other fungal species, an alignment of CreA and its homologues in Saccharomyces cerevisiae, Cryptococcus neoformans, Candida albicans, A. nidulans, A. fumigatus, Neurospora crassa, Trichoderma reesei, and Fusarium graminearum was carried out. Indeed, CreA homologues from filamentous fungi show phosphorylatable residues for all the sites identified by MS in the A. nidulans acidic and conserved domains except for S268, which was exclusively found in A. nidulans. Furthermore, identified residues, with the exception of S281, are either absent or have been replaced by nonphosphorylatable residues in the yeast species (Fig. 1b). The aim of this work was to characterize the effect of one specific phosphosite on CCR; hence, CreA double and triple point mutations were not investigated. Subsequently, phosphosites identified in this work and in previous studies were chosen for further characterization, as they are good representatives of phosphosites located in the acidic and conserved target phosphorylation hot spot regions of CreA. We chose S262 and S268 to be mutated to alanines, as these two residues were identified both *in silico* and *in vivo*, are located close to and within the acidic domain, and are present in filamentous fungi but not in yeast (Fig. 1b). Furthermore, S268 is unique to A. nidulans, which makes it a highly interesting target to investigate (Fig. 1b). Next, we chose a site located within the conserved region to be mutated. A total of 5 (S277, S281, S284, T308, and S312) sites were identified in the conserved region *in vivo*. We chose to mutate T308 to alanine, as this residue was identified *in silico* and *in vivo* (as opposed to S277 and S284), is located within the conserved region, and is present in both filamentous and yeast-like fungi. Although S312 is highly conserved between the here-analyzed species, this phosphosite was not characterized in this work because it, together with an additional site (S281) identified in different carbon sources, is the subject of another work in progress. Finally, we also included S319 in this analysis as a control, as this residue was previously identified as a phosphorylation target (28). CkiA is predicted to phosphorylate S262 and S268, and GskA is predicted to target T308 for phosphorylation. The site S319 has been experimentally confirmed to be the target of the protein kinase Stk22 (Sak1p homologue in S. cerevisiae, responsible for Snf1 activation [34]), which in turn is indirectly regulated by protein kinase A (PkaA). Mutation of these sites to an alanine mimics constant hypophosphorylation and allows us to determine the role of these phosphorylation sites for CreA function and CCR.

### CreA phosphomutations result in strains with defects in CCR.

Point mutations (S262A, S268A, T308A, and S319A) were generated in the CreA-GFP background, resulting in four strains. Strains S262A and S268A had the same growth phenotype as the wild-type (WT) strain, whereas strains T308A and S319A showed a dark green circle in the colony center compared to the WT strain in the presence of glucose (Fig. 1c). It is possible that these two sites are involved in developmental and conidiation processes, although growth fitness was not affected, with the exception of strain T308A, which grew significantly more (7%) than the WT strain at 37°C in minimal medium supplemented with glucose (see File S1 available at 10.6084/m9.figshare.13181990). Radial growth on plates of all strains was first assessed in the presence of the compounds allyl alcohol (AA) and 2-deoxyglucose (2DG), which are indicators for defects in CCR. AA is an alcohol that is metabolized to the toxic compound acrolein, and defects in CC derepression usually manifest themselves in strains with increased sensitivity to this compound (17, 25, 35). 2DG is a glucose analogue which, when metabolized, forms 2-deoxy-glucose-6-phosphate with the absence of the hydroxyl group on C-2 preventing further metabolism, resulting in its accumulation and inhibition of glycolysis (36). Strains with defects in CCR are more resistant to this compound and show reduced glucose uptake due to ability to metabolize different sugars in addition to glucose. Strains containing the point mutations S262A, S268A, and T308A were significantly more sensitive to 40 mM AA, while growth of the S319A strain was similar to that of the wild-type (WT) strain (Fig. 1d). Strains with the point mutations S262A and T308A were resistant to at least at one concentration of 2DG compared the resistance of the WT strain, whereas the S268A strain had similar growth to that of the WT strain (Fig. 1e). The strain containing the S319A mutation was significantly more sensitive to lower concentrations of 2DG but not to higher 2DG concentrations (Fig. 1e). These results suggest that the S262A and T308A mutations are important for CCR, whereas the strain with the S268A mutation showed a CCR-derepressing phenotype for AA only.

### CreA phosphomutations affect DNA binding.

**FIG 2:**
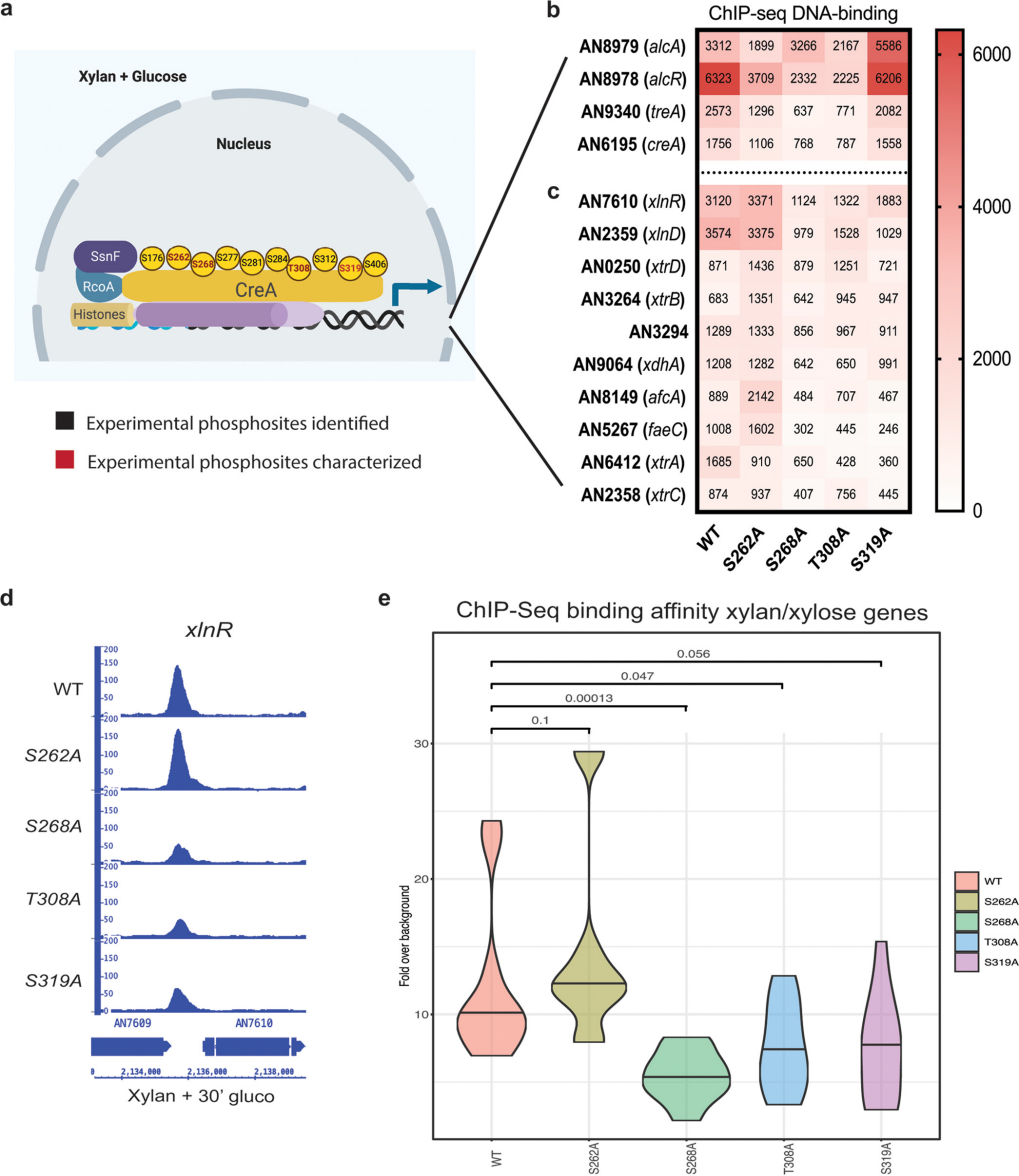
Summary of CreA phosphomutant phenotypes. (a) Diagram showing CreA protein localization and experimental phosphorylation sites identified by nLC-MS/MS, when the wild-type strain was incubated in minimal medium supplemented with 2% (wt/vol) glucose for 30 min. Experimentally identified CreA phosphosites are shown in black, and the four here-characterized phosphosites are shown in red. Heat map of CreA-GFP binding affinities, as determined by ChIP-seq under the same condition as described for panel a in the WT, S262A, S268A, T308A, and S319A strains showing *creA* and genes involved in alcohol and trehalose metabolism (b) and xylan/xylose metabolism (DNA-binding affinity average of three replicates is shown as a number in the center of each square) (c). (d) Integrative genome viewer of the *xlnR* promoter region showing reduced DNA-binding affinity in the S268A, T308A, and S319A strains. (e) Average CreA-GFP DNA-binding affinity, as identified by ChIP-seq, for all xylan/xylose genes in each CreA phosphomutation-harboring strain. Statistical analyses via two-way ANOVA multiple-comparison test using the WT as a reference.

In filamentous fungi, CreA regulates the expression of genes encoding enzymes required for alternative carbon source utilization, such as alcohol dehydrogenases (ADHs) and xylanases, through direct binding to the respective promoter regions (17, 18). We performed chromatin immunoprecipitation coupled to DNA sequencing (ChIP-seq) in order to determine whether the CreA phosphomutations impact CreA-GFP DNA binding dynamics in the simultaneous presence of xylan and glucose (induction of CCR and target promoter binding). Several experimental phosphosites were identified, among which, 4 representative phosphosites were chosen for point mutation, replacing the phosphosite with alanine for further ChIP-seq experiments (Fig. 2a). ChIP-seq results (NCBI accession number SUB7547091) show that CreA-GFP binds to several promoter regions of genes encoding enzymes required for ethanol, trehalose, and xylan/xylose utilization (Fig. 2b). In the presence of xylan and glucose, CreA-GFP bound to the promoter regions of the ADH *alcA* and the transcriptional regulator of alcohol metabolism *alcR*. The DNA binding to *alcA* was reduced in the S262A and T308A strains and increased in the S319A strain (Fig. 2b). Moreover, DNA binding in the S262A, S268A, and T308A strains was severely reduced at the promoter region of the TF *alcR*, the main regulator of alcohol metabolism and a known CreA target (37). Furthermore, DNA binding was also reduced in the S262A, S268A, and T308A strains for the trehalase-encoding gene *treA*, required for trehalose metabolism (Fig. 2b). Interestingly, *creA* is subject to self-binding and autoregulation, with strains harboring the phosphomutations S262A, S268A, and T308A showing significantly reduced binding (Fig. 2b).

In the presence of xylan and glucose, binding to the promoter regions of the TF XlnR, the xylanase-encoding gene *xlnD*, the xylose transporters XtrA to -D, the xylulose reductase XdhA, the alpha fucosidase AfcA, and the esterases FaeC and AN3294 was observed (Fig. 2c). Four (*xlnA*, *xlnB*, *xlnC*, and *xlnD*) of five of the A. nidulans xylanase-encoding genes have been shown to be under the transcriptional control of XlnR and of CreA-mediated CCR in a carbon source-dependent manner (18, 20, 38), whereas regulation of the fifth xylanase-encoding gene *xlnE* remains unknown. CreA-GFP with the S268A, T308A, and S319A phosphomutations had significantly reduced binding to the *xlnR* promoter region compared to that in the WT strain in the presence of xylan and glucose (Fig. 2c and d). In addition, CreA-GFP binding in the S268A, T308A, and S319A strains was also reduced for several promoter regions of genes involved in xylan/xylose metabolism (Fig. 2e). Conversely, binding of CreA-GFP in the S262A strain was increased compared to that in the WT strain for most of the xylan/xylose genes (Fig. 2c and e). In summary, CreA-GFP DNA-binding affinities to promoter regions of genes encoding enzymes required for ethanol, trehalose, and xylan/xylose utilization were altered in the phosphomutants. The strain S262A had similar binding as the WT strain for xylan/xylose genes and reduced binding to alcohol and trehalose metabolic genes. The mutants S268A and T308A consistently showed reduced binding to all here-investigated target DNA regions, whereas binding to xylan/xylose metabolic genes in the S319A strain was similar to that in the control strain. These results therefore suggest that targeted protein phosphomutation affects CreA TF DNA-binding affinities.

**FIG 3:**
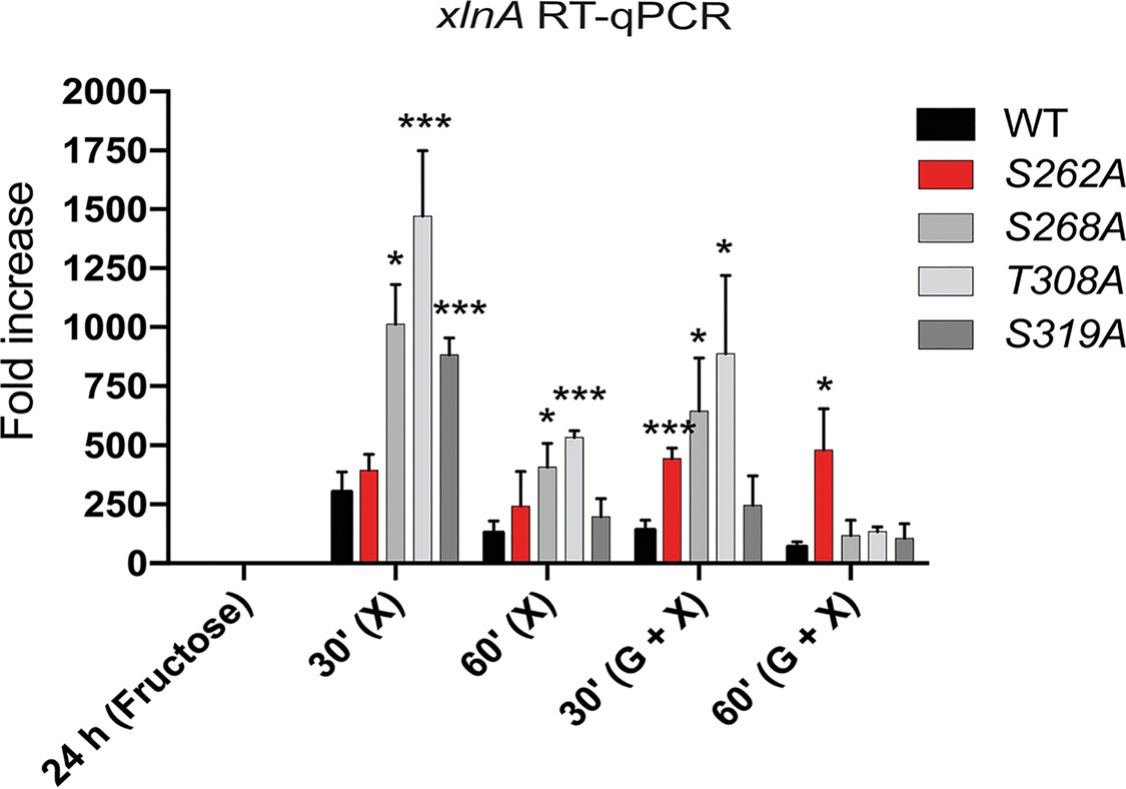
The expression of the xylanase-encoding gene *xlnA* is increased in the CreA phosphomutants. Strains were grown for 24 h in minimal medium (MM) supplemented with fructose and then transferred to MM supplemented with xylose or xylose and glucose for 30 min and 60 min. Total mRNA was extracted and reverse-transcribed to cDNA, and RT-qPCR was performed. Gene expression was normalized by the tubulin-encoding gene *tubC*. Standard deviations represent the averages from three biological replicates. *, *P* < 0.05; ***, *P* < 0.001 in a two-way ANOVA multiple-comparison test using the WT as a reference for each condition.

To support our ChIP-seq data, reverse transcription-quantitative PCR (qRT-PCR) studies on *xlnA* gene expression were performed in the presence of xylan or xylan and glucose. Results showed significant gene expression in the presence of glucose and xylan after 30 min in strains S262A, S268A, and T308A compared to that in the WT strain (Fig. 3). Similarly, *xlnA* expression was significantly increased in the presence of xylan in strains S268A, T308A, and S319A after 30 min and in strains S268A and T308A after 60 min compared to that in the WT strain (Fig. 3). In addition, strain S262A showed increased expression of *xlnA* in the presence of glucose and xylan after 60 min of treatment (Fig. 3). These results suggest that the CreA phosphomutations impact transcriptional expression of *xlnA*. Furthermore, *xlnA* expression in the presence of xylan and glucose does not always reflect the ChIP-seq data, with binding to the *xlnR* promoter not being affected in the S262A strain. In the S319 strain, *xlnA* expression was not different from that in the WT strain, although CreA-GFP binding was significantly reduced in this strain. In contrast, *xlnA* expression was significantly induced in the S268A and T308A strain after a 30-min incubation in xylan and glucose, which agrees with the ChIP-seq data, where significantly reduced binding to the *xlnR* promoter region was observed. These results suggest that additional mechanisms, other than DNA binding, exist and impact CreA-mediated CCR, including competition between transcription factors for promoter target site binding. The pH-responsive TF PacC has been shown to indirectly regulate *xlnA* expression, and two PacC-binding consensus sequences are present in the *xlnA* promoter region, although their functionality has not been tested to date (39).

### CreA phosphomutations affect CreA-GFP cellular localization.

**FIG 4:**
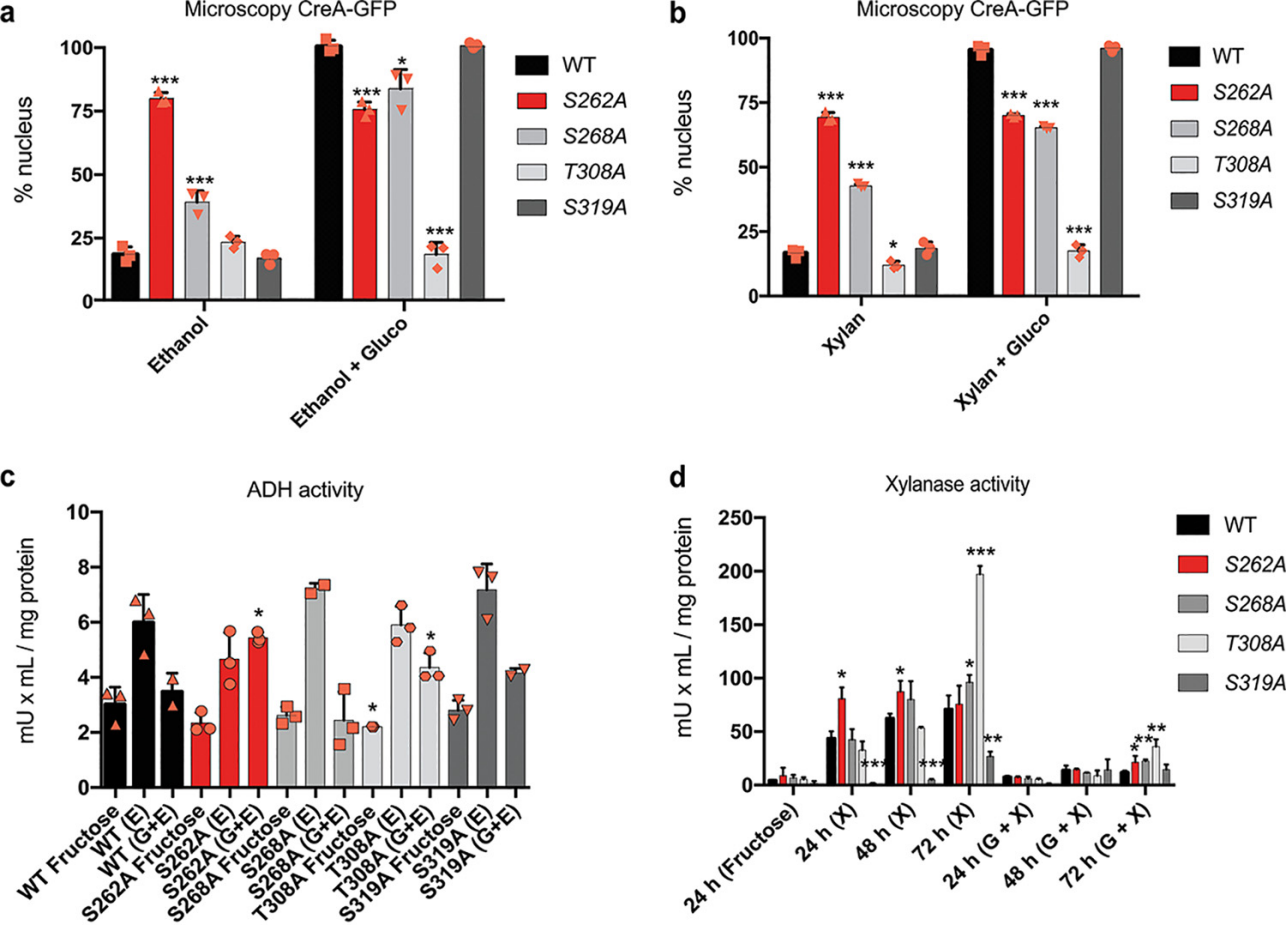
CreA phosphomutations affect cellular localization and enzyme activities. Percentages of nuclear CreA-GFP localization, as determined by fluorescence microscopy of strains grown for 16 h at 22°C in minimal medium (MM) supplemented with ethanol (a) or xylan (b) and after the addition of 2% glucose for 30 min. Nuclei were counted for at least 100 hyphal germlings and stained with Hoechst 33258 to confirm GFP fluorescence localization. Percentage represents nuclear CreA-GFP in comparison to total counted nuclei. (c) Alcohol dehydrogenase (ADH) activity is increased in the S262A and T308A phosphomutants in the presence of ethanol and glucose. Strains were grown in MM supplemented with fructose (noninducible) for 24 h and then transferred to MM supplemented with ethanol and glucose or ethanol only for 2 h. ADH activity was measured in mycelial protein extracts and normalized by total intracellular protein. (d) Xylanase activity is impaired in the phosphomutants. Strains were initially grown as described for panel c and then transferred to MM supplemented with xylose and glucose or xylose for 24 h, 48 h, and 72 h, before xylanase activity was measured in culture supernatants. Standard deviations represent the averages from three biological replicates (shown as orange symbols). *, *P* < 0.05; **, *P* < 0.01; ***, *P* < 0.001 in a two-way ANOVA multiple-comparison test using the WT (wild-type) strain as a reference for each condition.

A possible additional mechanism that controls CCR is the cellular localization of CreA-GFP. The presence of glucose causes CreA to translocate to the nucleus where it causes gene repression (3). To determine whether the phosphomutations affect CreA cellular localization under both CC-derepressing and CCR conditions, strains were grown for 16 h in minimal medium (MM) supplemented with 1% ethanol or 1% xylan (CC derepression). After the addition of 2% glucose (to induce CCR) and further incubation for 30 min, microscopy was carried out. In agreement with other studies (3, 13, 24), around 20% of WT CreA-GFP was nuclear in the presence of ethanol and xylan, while the addition of glucose caused 100% nuclear localization in this strain (Fig. 4a and b). Strains containing the S262A and S268A mutations showed increased CreA-GFP nuclear localization under derepressing conditions compared to that in the WT strain (Fig. 4a and b). In contrast, the other two phosphomutations had no significant effect on CreA-GFP cellular localization under these conditions, with the exception of the T308A strain, which showed significantly reduced nuclear localization in the presence of xylan (Fig. 4a and b). Upon the addition of glucose, nuclear localization of the strain containing the S262A mutation did not change compared to that under the CC-derepressing condition and was significantly lower than that observed for the WT strain, suggesting that this mutation “keeps” CreA-GFP locked in the nucleus, affecting the ability to move out (Fig. 4a and b). The mutation S268A resulted in increased nuclear accumulation of CreA-GFP in the presence of glucose, albeit to significantly lower levels than in the WT strain, suggesting that this mutation is also important for CreA-GFP cellular localization under CCR conditions (Fig. 4a and b). The nuclear localization of CreA-GFP in the T308A strain in the presence of glucose was similar to that under CC-derepressing conditions and significantly reduced compared to that in the WT strain, suggesting that this mutation is required for CreA translocation and/or accumulation in the nucleus (Fig. 4a and b). In contrast, cellular localization patterns for the S319A mutation-harboring strain were similar to those for the WT strain.

### CreA phosphomutations affect enzyme activities for alternative carbon source utilization.

The aforementioned results show that CreA phosphomutations affect DNA-binding affinity, target gene expression, and cellular localization. To determine the impact on the proteins, we assayed the activities of alcohol dehydrogenase (ADH) and xylanases, whose genes are known to be transcriptionally regulated by CreA (17, 18, 40–42), in the point mutation-harboring strains. Strains were first grown in MM supplemented with fructose (noninducing, nonrepressive carbon source) and mycelia were subsequently transferred to MM supplemented with ethanol (ADH induction), ethanol and 2% glucose, xylan, or xylan and 2% glucose. ADH activity increased in the presence of ethanol in the WT strain and was at a basal level in the presence of glucose (Fig. 4c). ADH activity in the strains harboring the S268A and S319A mutations was similar to that in the WT strain (Fig. 4c). In contrast, ADH activity in the presence of ethanol and glucose in the S262A-harboring strain was similar to that in the WT strain in the presence of only ethanol, suggesting that this mutation inhibits CC-induced repression of ADH (Fig. 4c). Lastly, ADH activity in the T308A-harboring strain was reduced in the control condition and significantly increased in the presence of ethanol and glucose but did not differ from that in the WT strain in the presence of ethanol, again suggesting that glucose-mediated CCR did not result in ADH transcriptional repression in this strain (Fig. 4c).

Next, we quantified xylanase activities of culture supernatants every 24 h over 72 h due to xylan being a complex carbon source, which, when degraded, results in a gradual release of enzyme-inducing mono- and polysaccharides (43). In agreement with other studies (13, 24), xylanase activity was at a basal level in the presence of xylan and glucose in the WT strain but increased with prolonged incubation times, due to the complete consumption of available extracellular glucose (Fig. 4d). In the WT strain, xylanase activity was much higher in the presence of xylan than in the presence of xylan and glucose and also increased with prolonged incubation times (Fig. 4d). In the simultaneous presence of glucose and xylan, strains harboring the S262A, S268A, and T308A mutations, but not the S319A mutation, had significantly increased xylanase activity after 72 h compared to that in the WT strain (Fig. 4d), suggesting defects in CCR. In the presence of xylan, the S262A strain had significantly increased xylanase activity after 24 h and 48 h compared to that in the WT strain (Fig. 2d). It should be noted that enzyme activity in this strain remained the same at all tested time points, and no gradual increase in activity was observed, suggesting CC derepression at all tested time points. The strains S268A and T308A presented significantly increased xylanase activity after 72 h in the presence of xylan and glucose compared to that in the WT strain (Fig. 4d). In contrast, xylanase activity in culture supernatants of the S319A strain in the presence of xylan was significantly reduced at all the assayed time points (Fig. 4d), suggesting that this mutation affects CC derepression.

In summary, these CreA phosphomutations affect CreA cellular localization and cause defects in CCR and CC derepression. The S262A mutation results in a strain that is blind to glucose as a carbon source, as CreA nuclear localization did not change between CCR and CC-derepressing conditions. Similarly, the S268A phosphomutation causes increased CreA nuclear localization under CC-derepressing conditions, decreased nuclear localization under CCR conditions, and xylanase activity that is significantly higher under all conditions, suggesting that this mutation also impairs glucose utilization and/or signaling. The T308A mutation mainly affects CCR, with CreA being excluded from the nucleus and enzyme activities being significantly increased in the presence of glucose and at the latest incubation time points. Lastly, the S319A mutation does not impact CreA cellular localization and ADH activity, whereas xylanase activity was significantly reduced, suggesting that this mutation does not impact transcriptional regulation (similar *xlnA* levels and binding affinity to xylan-encoding genes compared to those for the WT strain) of xylanase-encoding genes, but rather impacts a region of CreA that is important for xylanase secretion and/or translation.

### CreA phosphomutations affect protein accumulation.

**FIG 5:**
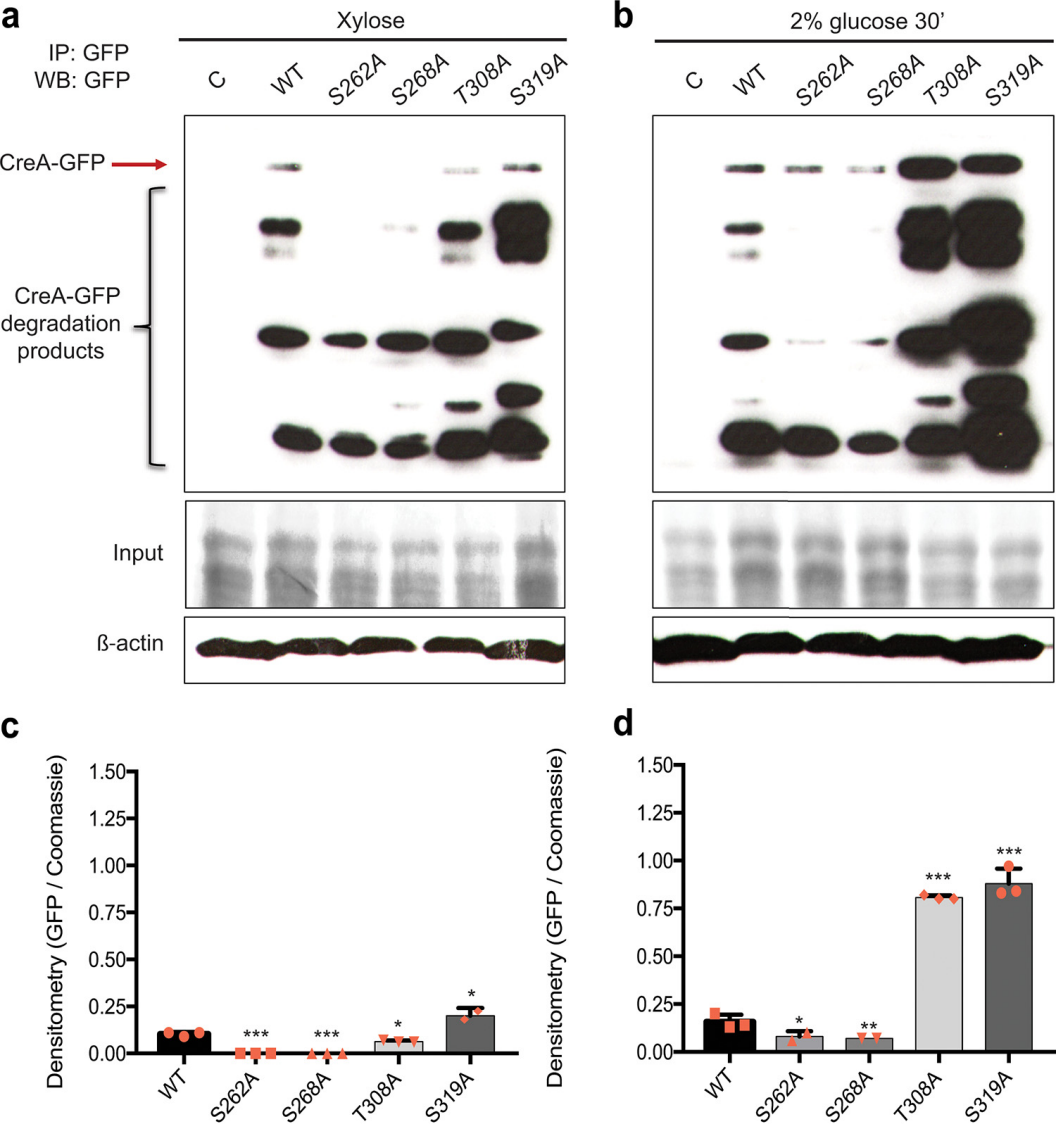
CreA phosphomutations affect protein accumulation. Strains were grown in minimal medium (MM) supplemented with 1% (wt/vol) xylose for 24 h before 2% (wt/vol) glucose was added for 30 min. Total cellular proteins were extracted, and CreA-GFP was immunoprecipitated. Samples were run on a protein gel and Western blotting was carried out (top). The red arrow indicates full-length CreA-GFP in the presence of xylose (a) and xylose and glucose (b). The bottom shows the total protein input used for immunoprecipitation as stained with Coomassie blue. Furthermore, Western blotting of total protein extract for β-actin antibody was performed and used for normalization. (c and d) Graphs representing densitometry scans of the Western blots described in panels a and b, respectively. Densitometry values for CreA-GFP were normalized by the input. Standard deviations represent the averages from three biological replicates (shown as orange symbols). *, *P* < 0.05; **, *P* < 0.01; ***, *P* < 0.001 in a two-way ANOVA multiple-comparison test using the WT as a reference for each condition.

Based on the aforementioned results, we turned our attention to posttranslational mechanisms which have been shown to also be involved in the regulation of CreA function. A previous study has shown that CreA partially relies on *de novo* synthesis and that low cytoplasmic levels of this protein are always present, thus allowing the fungus to rapidly respond to and scavenge for extracellular glucose concentrations (13). To determine whether the phosphomutations affect CreA protein levels under both CC-derepressing and CCR conditions, the WT, S262A, S268A, T308A, and S319A strains were grown in MM supplemented with xylose for 24 h before 2% glucose was added for 30 min, immunoprecipitation was carried out on whole-cell protein extracts, and Western blotting was performed using an anti-GFP antibody. In agreement with previous studies (13, 24), CreA protein levels increased upon the addition of glucose in the WT strain. This increase was also observed for the strains harboring the CreA point mutations, although protein concentrations differed significantly compared to those in the WT strain (Fig. 5). The S262A, S268A, and T308A strains presented significantly reduced CreA protein levels under CC-derepressing conditions compared to those in the WT strain (Fig. 5a and c). In the S319A strain, total CreA protein levels were significantly increased under all conditions compared to those in the WT strain (Fig. 5), which is in agreement with a previous study (28). Strains harboring the S262A and S268A mutations also presented significantly reduced CreA protein levels in the presence of glucose compared to that in the WT strain (Fig. 5b and d). In contrast, the strain harboring the T308A mutation presented CreA protein levels that were significantly increased in the presence of glucose compared to that in the WT strain (Fig. 5b and d). Our ChIP-seq analysis showed that CreA-GFP binds to its own promoter region, suggesting autoregulation (Fig. 2b). Binding affinities were similar between the WT and S319A strains but significantly reduced in the S262A, S268A, and T308A strains (Fig. 2b). Additional posttranslational mechanisms of self-regulation are likely to exist, as CreA-GFP protein accumulation was similar in strains T308A and S319A, whereas DNA binding was not. Further studies are required to elucidate the mechanism on how CreA stability is regulated. These results indicate that the CreA phosphomutations affect CreA protein concentrations.

### CreA phosphomutations result in a dysregulated glycogen and trehalose metabolism.

**FIG 6:**
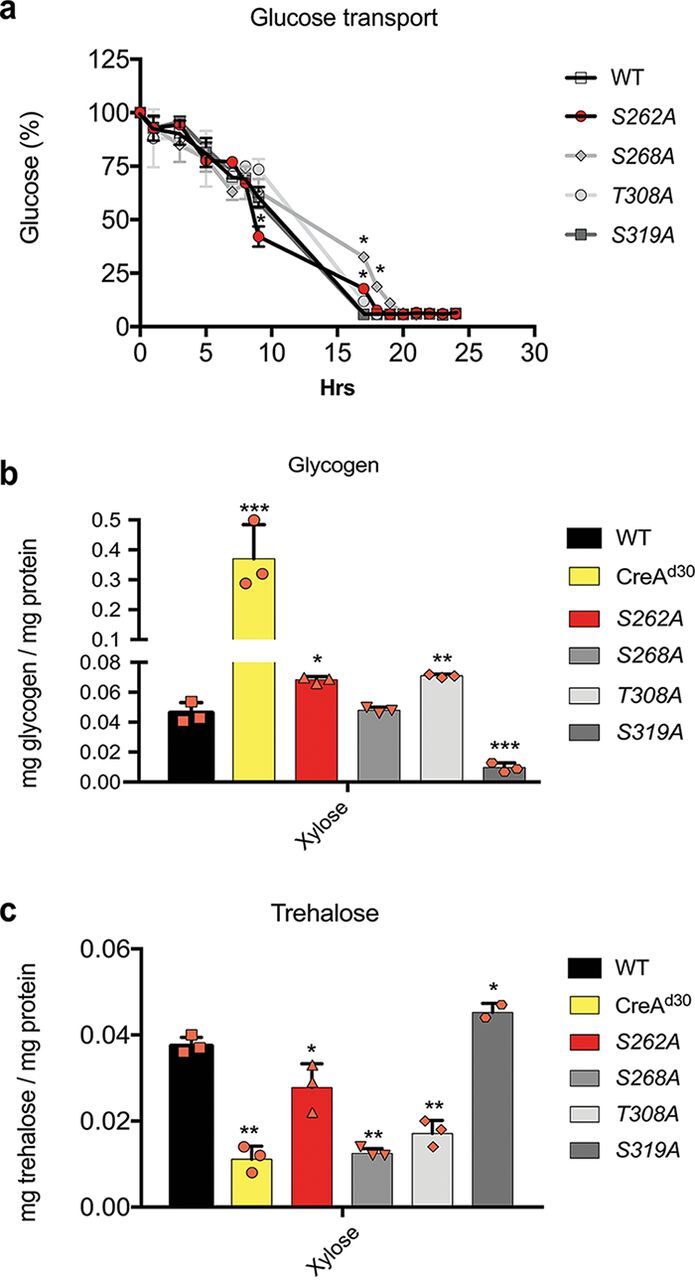
CreA phosphomutations affect glycogen and trehalose metabolism. (a) Glucose transport is not impaired in the CreA-GFP phosphomutants. Strains were grown for 24 h in complete medium and then transferred to minimal medium (MM) supplemented with glucose for 24 h. Supernatant samples were collected at the indicated time points, and glucose concentrations were measured using the glucose oxidase kit God-PAP. (b) CreA controls glycogen metabolism. Strains were grown in MM supplemented with xylose for 24 h before glycogen was extracted and digested with amyloglucosidase, and the concentration of free glucose was measured as described for panel a. The *creA*^d30^ strain was used as a control. (c) CreA controls trehalose metabolism. Strains were grown under the same conditions as described for panel b, before total cellular protein was extracted and 5 μg was used to quantify intracellular trehalose concentrations using the Megazyme trehalose assay kit. Standard deviations represent the averages from three biological replicates (shown as orange symbols). *, *P* < 0.05; **, *P* < 0.01; ***, *P* < 0.001 in a two-way ANOVA multiple-comparison test using the WT as a reference for each condition.

Glycogen metabolism has also been shown to be under the regulatory control of CreA and its homologues in several fungi, with the lack of CreA causing an accumulation of intracellular glycogen (14–16). In A. fumigatus, CreA is required for the expression of *gdbA*, encoding the glycogen debranching enzyme, and in the absence of CreA, intracellular glycogen levels increase due to the activity of glycogen synthase and the absence of GdbA (14). Unexpectedly, our ChIP-seq analysis showed no binding of A. nidulans CreA-GFP to the *gdbA* promoter region, indicating possible indirect regulation of glycogen synthesis by CreA. To determine whether glycogen metabolism was impaired in strains harboring the CreA point mutations, the glycogen levels were used as a reporter in an assay checking indirect CreA function. We first quantified glucose transport in these strains, as glucose is the precursor in glycogen biosynthesis. Strains were grown in complete medium for 24 h before being transferred to MM supplemented with 1% glucose for 24 h. Glucose concentrations were measured in culture supernatants at different time points. The WT strain consumed all extracellular glucose after 16 h, and glucose transport did not differ significantly between strains for the first 10 h. After 17 h and 19 h, extracellular glucose remained in culture supernatants of strains harboring the S262A and S268A mutations, suggesting that these strains were delayed in glucose transport (Fig. 6a). These results suggest that glucose transport is not impaired in the CreA mutation strains. Next, we measured intracellular glycogen concentrations when the WT, S262A, S268A, T308A, and S319A strains were grown in MM supplemented with xylose for 24 h, the condition when glycogen is required as a carbohydrate storage compound, providing a carbon source to the fungus. We used the CreA^d30^ strain, which carries a truncation of CreA (9) to show lack of function of CreA. In agreement with results in the A. fumigatus Δ*creA* strain, truncation of A. nidulans *creA* resulted in an accumulation of intracellular glycogen that was almost 10-fold higher than the glycogen concentrations in the WT strain (Fig. 6b). The S262A and T308A strains also had significantly increased glycogen levels, although not to the same concentration as the CreA^d30^ strain, whereas the S319A strain had significantly reduced intracellular glycogen levels compared to those in the WT strain (Fig. 6b). The S268A point mutation did not affect intracellular glycogen concentrations. These results suggest that A. nidulans CreA is also involved in the regulation of glycogen metabolism and that the CreA phosphomutations affect the function of CreA.

In A. fumigatus, the inability to use glycogen, such as cell wall polysaccharides, as a precursor carbon source for cellular metabolic pathways results in the fungus using trehalose, which is another major intracellular carbohydrate storage compound (14). Our ChIP-seq results already showed that CreA binds to the promoter region of *treA*, coding for an enzyme involved in trehalose metabolism (Fig. 2b). The S262A, S268A, and T308A strains showed reduced DNA-binding affinity for *treA* compared to that of the wild-type strain (Fig. 2b). To determine whether trehalose metabolism is affected by our CreA point mutations, we measured intracellular trehalose concentrations under the same conditions as described above. In agreement with reference 9 and studies in A. fumigatus, the A. nidulans CreA^d30^ strain had significantly reduced intracellular trehalose levels (Fig. 6c). Consistent with our ChIP-seq data, strains harboring the CreA point mutations S262A, S268A, and T308A also had significantly decreased intracellular trehalose concentrations compared to that in the WT strain, whereas the strain with the S319A mutation had significantly increased trehalose levels (Fig. 6c). In summary, these results suggest that the S262A and T308A point mutations impair CreA function for the regulation of glycogen and trehalose metabolism. Furthermore, glycogen and trehalose levels were the opposite in the S319A point mutation strain compared to those in the CreA^d30^ strain, suggesting that this mutation may increase the CreA repressive regulatory control of glycogen and trehalose metabolism.

## DISCUSSION

The main regulator of CCR in biotechnologically relevant fungi is a C_2_H_2_-type TF, which is termed CreA in the reference fungus A. nidulans. Regulation of CreA itself is highly complex and involves protein-protein interactions and posttranslational modifications which ultimately control CreA cellular localization and protein turnover dynamics (13, 24). Although posttranslational modifications have been shown to occur on fungal CreA homologues (23, 44–46), in-depth studies describing these modifications have not been reported to date. This study attempts to characterize CreA posttranslational events, including sumoylation, ubiquitylation, and phosphorylation, under the condition where CCR occurs. Sumoylation and ubiquitylation were not identified on CreA. It is still possible that these posttranslational events occur on CreA but under other conditions, such as shorter or prolonged incubation times in glucose or in the presence of other carbon sources. Alternatively, these modifications could occur on CreA protein interaction partners, resulting in the degradation of the whole complex. Previous work has shown that ubiquitylation is required for CreA degradation processes in the absence of glucose (13); however, the CreB deubiquitinating enzyme does not directly act on CreA, and it is involved in CCR regulation (47). The ubiquitylation process is likely to occur on proteins which form a complex with CreA. Detecting the presence of additional posttranslational modifications on CreA under different conditions remains a subject for future investigations.

In contrast, several phosphorylation sites were detected on CreA under conditions that induce CCR, indicating that this posttranslational modification is important for CreA regulation in the presence of glucose. The detection of CreA phosphorylation agrees with studies on S. cerevisiae Mig1p (CreA homologue), Aspergillus oryzae CreA, and *T. reesei* CRE1. In S. cerevisiae, the absence of glucose causes the phosphorylation and activation of the protein kinase Snf1p, which subsequently translocates to the nucleus; in the nucleus, Snf1p phosphorylates Mig1p, causing Mig1p to unbind from target DNA sequences and to translocate to the cytoplasm, thus alleviating Mig1p-mediated repression of target genes (21). In A. oryzae, phosphorylation in the C-terminal region affects CreA cellular localization and degradation (23), whereas phosphorylation of *T. reesei* CRE1 serine 241 is crucial for CRE1 DNA binding in the presence of glucose (30). Previous work has shown that Stk22 (a kinase working as an activator of SnfA) is regulated by PkaA and acts on CreA (28). In A. nidulans, SnfA is not predicted to target CreA for phosphorylation, and SnfA has never been identified as a CreA interaction partner (24). On the other hand, CreA requires SnfA for normal localization in the cytoplasm (3). These studies suggest that SnfA regulates CreA indirectly through an as-yet-unknown mechanism.

The phosphorylation sites identified by MS in this work, with the exception of CreA S277 (predicted to be phosphorylated by PkaA) and S406 (predicted to be phosphorylated by an unspecific protein kinase), were predicted *in silico* to be phosphorylated (Fig. 1a), indicating that the bioinformatics tool NetPhos is a good starting point for detecting potential phosphorylation sites on *Aspergillus* proteins. The acidic and conserved domains show an increased concentration of phosphorylation sites, suggesting that these regions are important for glucose-mediated CCR. Deletion of these CreA regions has been shown to affect CreA cellular localization and growth in the presence of different carbon sources (13). In agreement, mutation of S262, S268, T308, and S319 to alanines also resulted in strains with defects in CreA cellular localization (with the exception of S319, which shows a delay in CreA transport to the nucleus [28]), DNA-binding affinity, and protein accumulation, with consequences for the regulation of enzyme activities and glycogen and trehalose metabolism (Fig. 2 and 6). Interestingly, the S319A point mutation, which localizes outside the acidic and conserved domains, resulted in a strain that showed different, often opposing, phenotypes than the strains harboring the S262A, S268A, and T308A mutations, which are located around the acidic and conserved domains. For example, binding to the *xlnR* promoter region was reduced in the S319A strain, although xylanase activity was not affected as was observed for the other phosphomutation-harboring strains. It is possible that phosphorylation sites located within or close to the acidic and conserved domains are important for structural organization and function of CreA. Phosphosites may also affect protein-protein interactions and subsequent signaling events, resulting in phenotypes that cannot be explained by DNA-binding capacity alone. This is further supported by our findings where differences in CreA-GFP-binding affinities to the *creA* promoter region do not necessarily reflect subsequent cellular protein levels. Another intriguing possibility is that different parts of the protein have separate functions, which depend on the condition-specific phosphorylation pattern. Again, CreA posttranslational events need to be further characterized under other biotechnologically relevant conditions. In addition, future studies that include protein-protein interactions of CreA phosphomutants will also be highly informative on the regulatory processes underlying CCR.

Economically feasible conversion of plant biomass to simple sugars by filamentous fungi requires the attenuation or absence of CCR in order to ensure continuous enzyme secretion (48). Regulation of CCR necessitates the expression of protein-encoding genes that are essential for fungal survival and growth and can therefore not be deleted, whereas promoter replacement of these essential genes is often leaky and inefficient (49). Mutation of phosphorylation sites, as described in this work, is an alternative for attenuating CCR and increasing enzyme secretion in the presence of glucose without affecting fungal growth.

In this study, we show that the absence of CreA phosphorylation sites affect protein concentrations under both CC-derepressing and CCR conditions. The phenotypes observed for each point mutation-harboring strain are summarized in Table 1. The S262A and S268A mutations cause a significant reduction of CreA protein levels under all conditions (Fig. 5). Accordingly, we observed an increase in xylanase gene expression (Fig. 3) and enzyme activities (Fig. 4c and d) as well as intracellular glycogen and trehalose levels that are similar to those in the *creA*^d30^ strain (Fig. 6b and c), suggesting that these mutations inactivate CreA. In contrast, CreA nuclear localization is increased in the S262A and S268A strains under CC-derepressing conditions. In the S262A strain, we observed a DNA binding affinity for xylan/xylose genes similar to that in the WT strain, despite this strain presenting reduced CreA protein levels, thus resulting in increased xylanase activity (Fig. 2e, 4d, and 5). These results highlight the necessity to carry out both transcriptional and posttranslational characterizations. Similarly, CreA nuclear accumulation was increased in the S268A strain, whereas DNA-binding affinity for xylan/xylose genes was reduced, again resulting in an increase in enzyme activities (Fig. 4c and d). Both the S262 and S268 phosphosites are predicted to be phosphorylated by the essential protein kinase CkiA, and indeed, when placing *ckiA* under the thiamine-repressible promoter, a CC-derepressing phenotype is observed (24).

**TABLE 1:** Phenotype summary

Phenotype	Value or description for phosphosite:				
	WT	S262A	S268A	T308A	S319A
Phosphosite conservation^*a*^		F (all tested)	F (A. nidulans)	F and Y (*C. neoformans*, C. albicans)	F (A. nidulans, T. reesei, F. graminearum)
Radial growth, allyl alcohol (40 mM) (%)	44.68	8.96	10.02	10.64	54.76
Radial growth, 2DG (0.25 mM) (%)	33.67	38.1	32.6	49.57	34.04
Nuclear CreA-GFP (glucose) (%)	93.52	70.2	77.9	16.41	93.88
ADH activity (glucose+ethanol) (mU × mg/mg protein)	3.47	5.43	2.43	4.35	4.15
RT-qPCR for *xlnA* (30-min glucose+xylose) (fold increase)	140.11	443.35	646.46	886.86	246.05
Xylanase activity (72-h glucose+xylose) (mU × mg/mg protein)	11.82	21.43	22.38	35.9	14.56
Stability of CreA protein (30-min glucose) (GFP/Coomassie ratio)	0.16	0.08	0.07	0.81	0.88
Glucose supernatant (%) (17-h transport)	5.74	17.77	32.56	11.96	5.74
Glycogen (mg/mg of protein)	0.046	0.068	0.048	0.071	0.01
Trehalose (mg/mg of protein)	0.038	0.028	0.013	0.017	0.045

a F, filamentous fungi; Y, yeast.

In the S319A strain, xylanase activity was significantly reduced despite no changes in CreA nuclear localization and reduced binding affinities for genes encoding enzymes required for xylan degradation. This discrepancy is possibly due to the high CreA protein levels observed in the S319A mutant (Fig. 5). Similarly, CreA cellular localization patterns, DNA-binding affinities, and glycogen and trehalose concentrations as well as enzyme activities are also not in agreement with each other in the T308A strain. This mutant showed high CreA protein levels in the presence of glucose; however, the T308 mutant has reduced DNA binding and nuclear localization of CreA, events that together result in a CC-derepressing phenotype. This point mutation is predicted to be phosphorylated by the protein kinase GskA. GskA-GFP studies showed a predominant cytoplasmic localization (24), suggesting that CreA phosphorylation by GskA takes place in the cytoplasm and that this event is required for CreA translocation into the nucleus (Fig. 4a and b). Pharmaceutical inhibition of GskA by GSK3β-VII strongly suggests involvement of this protein kinase in CCR via regulation of the mitogen-activated protein kinase kinase (MAPKK) PbsA (50). These results highlight the regulatory complexity at both transcriptional and posttranslational levels that govern CreA, suggesting additional mechanisms which probably occur posttranslationally.

We also attempted to replace the S262, S268, T308, and S319 residues with glutamic acids in order to mimic constant phosphorylation but were unable to do so, even in a diploid strain carrying a heterozygous mutation resulting in a very sick and unviable strain, suggesting that these mutations result in a lethal dominant phenotype. Indeed, in Candida albicans, hyperactivation of Mig1p due to Snf1 deletion resulted in a lethal dominant phenotype, suggesting that correct levels of this regulator and DNA binding are important for fungal survival (51).

**FIG 7:**
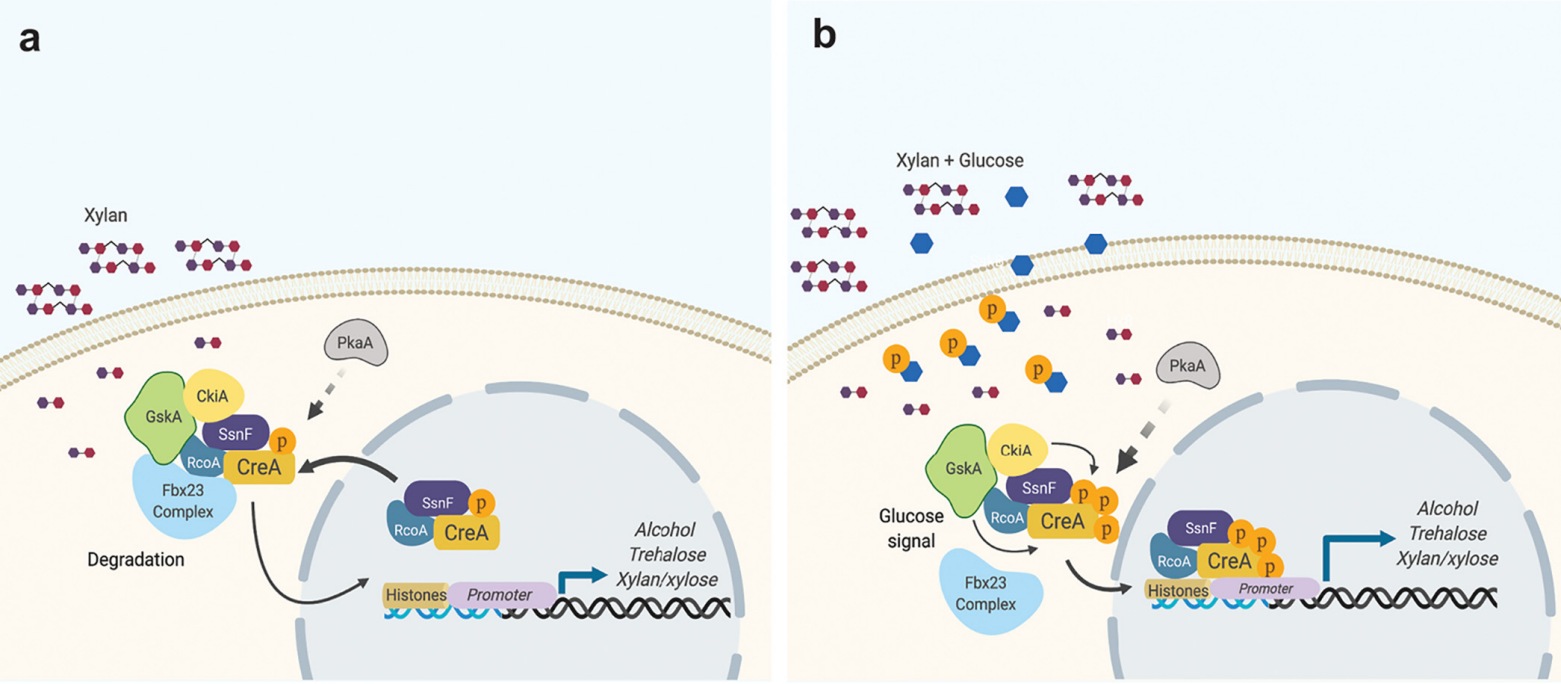
Diagram showing the phosphorylation events that occur in the presence of glucose. (a) CreA forms a cytoplasmic complex with the corepressors RcoA and SsnF, the protein kinases GskA and CkiA, and the F-box protein Fbx23 in the presence of alternative carbon sources, such as xylan (24). Under these conditions, CreA is also present in the nucleus with the corepressors, although DNA binding is not occurring. PkaA is also involved indirectly in the process, regulating CreA stability and localization (1, 28, 50). (b) Glucose is taken up into the cells, where it undergoes glycolysis and phosphorylation, a step that has been shown as a crucial signal for CreA translocation to the nucleus. Upon glucose signal detection, Fbx23 dissociates from the complex, CreA phosphorylation increases, and it translocates to the nucleus without the protein kinases. In the nucleus, CreA binds to target genes and causes repression.

Under CC-derepressing conditions, CreA is localized in the cytoplasm and the nucleus (1, 3, 13, 24), with cytoplasmic CreA forming a complex with Fbx23, which signals degradation of these proteins (Fig. 7a). Although CreA posttranslational modifications remain to be fully characterized under CC-derepressing conditions, it is likely that little phosphorylation occurs on this protein under these conditions, as suggested previously (29). Upon the addition of glucose, several phosphorylation events, which are catalyzed by different protein kinases, occur on the CreA acidic and conserved domains, resulting in CreA translocation to the nucleus, binding to target gene promoter regions such as *alcA*, *alcR*, *treA*, and *xlnR* promoters, and induction of CCR (Fig. 7b). Another protein kinase involved in this process is PkaA (catalytic subunit of PKA), previously shown to be required for CreA stability (50). ChIP-seq under CCR found CreA-GFP binding to the *xlnR* and *xlnD* promoters and additional genes involved in xylan/xylose metabolism (Fig. 2b and c). Previous work showed that although *xlnD* is under the control of CreA, its expression is partially controlled by XlnR (18). Our results showed that CreA binds directly to the *xlnR* and *xlnD* promoters (Fig. 2c), explaining *xlnD* derepression when CreA function is affected and *xlnA*, *xlnB*, and *xlnC* derepression (controlled by XlnR) (18, 48, 52).

The here-described phosphomutant-associated phenotypes can serve as starting points for strain engineering in order to modify CreA without the need to carry out whole gene deletions in biotechnologically relevant fungi and for studying CreA homologues in plant and human-pathogenic fungi, where it was shown to be important for the infection process (51, 53–55). In summary, our results indicate that CreA is regulated by posttranslational events that are predicted to be controlled by different protein kinases. The exact regulatory mechanism exerted by these enzymes remains a subject for future investigations, but conservation of these phosphorylation sites in a number of fungi as well as a functional conservation of this TF in these organisms suggests similar regulatory mechanisms.

## MATERIALS AND METHODS

### Strains and culture conditions.

Strains used in this study are described in Table S2 available at 10.6084/m9.figshare.13181990. GFP-tagged CreA and CreA point mutations were constructed in the AGB551 background strain as described previously by de Assis et al. (24). Briefly, point mutations in CreA were introduced by generating two fragments by PCR, using the CreA-GFP strain as a template, for cassette construction: (i) the 5′ UTR (untranslated region) and the CreA open reading frame (ORF) spanning until 3 bp before the point mutation and (ii) the rest of the CreA ORF, which overlaps 20 bp with the 5′ UTR-CreA ORF and contains three different base pairs for amino acid substitutions in the *gfp* gene, the marker gene, and the 3′ UTR. Fragments were ligated using S. cerevisiae strain SC9741 and plasmid pRS426 as described previously (13). Cassettes were amplified by PCR from positive candidates by using the TaKaRa high-fidelity polymerase (Clontech) and subsequently transformed into A. nidulans as described previously (24). Strains were confirmed by sequencing. Strain CreA^d30^ was kindly provided by M. Orejas. All culture medium and growth conditions were as described previously (1, 13).

### CreA phosphorylation site prediction and protein alignment.

Phosphorylation sites on CreA were predicted by submitting the CreA protein sequence (retrieved from FungiDB) to NetPhos3.1 (http://www.cbs.dtu.dk/services/NetPhos/) and using the default filters. Significant predicted phosphorylation sites were considered those having a score higher than 0.5 (31, 32). Alignment between CreA and its homologues from other fungi was carried out using SnapGene multiple-sequence alignment software and analyzed at ESPript 3.0 (http://espript.ibcp.fr/ESPript/cgi-bin/ESPript.cgi). All protein sequences were obtained from the UniProt database (https://www.uniprot.org).

### Growth in the presence of 2-deoxyglucose and allyl alcohol.

Strains were grown from 10^7^ conidia on minimal media (MM) (1) supplemented with 1% (wt/vol) xylose or glucose and increasing concentrations of 2DG or AA, respectively, for 5 days at 37°C or 30°C. Colony diameters were measured from biological triplicates.

### Microscopy.

Strains were grown for 16 h at 22°C in 5 ml MM supplemented with 1% (wt/vol) xylan or ethanol in small petri dishes containing a coverslip before glucose was added to a final concentration of 2% (vol/vol). Samples were then incubated an additional 30 min. Coverslips containing the attached hyphal germlings were viewed under a Carl Zeiss (Jena, Germany) Axio Observer Z1 fluorescence microscope using the 100× oil immersion lens objective (EC Plan-Neofluar, numerical aperture [NA] 1.3). Differential interference contrast (DIC) and fluorescence images were taken and processed, and the percentage nuclear CreA-GFP was calculated as described previously (13).

### Alcohol dehydrogenase activity.

ADH activity was determined as described previously (56). Briefly, total cellular proteins were extracted from strains grown under the defined conditions, and 10 μg of total cellular protein was used to measure ADH activity in a total reaction volume of 200 μl. Proteins were first mixed with reaction buffer (50 mM sodium pyrophosphate decahydrate, 50 mM semicarbazide hydrochloride, and 20 mM glycine, pH 8.0) before the reaction was started with 6 mM NAD^+^ and incubated at 37°C for 15 min. Enzyme activity was measured at 340 nm in a 96-well plate using a Synergy HXT microplate reader (Biotek). The linear part of the slope was used to calculate enzyme activity, which was expressed as milliunits × milliliters per milligram protein. ADH activity was measured in biological and technical triplicates.

### Xylanase activity.

Xylanase (endo-1,4-β-xylanase) activity in culture supernatants of biological triplicates was performed using Azo-Xylan from birch-wood (Megazyme) as a substrate according to the manufacturer’s instructions. Technical triplicates were carried out for each biological replicate.

### Chromatin immunoprecipitation followed by next-generation sequencing and bioinformatics analysis.

The AGB551 (GFP-free, control WT strain) and CreA-GFP phosphomutation-harboring strains were cross-linked in 1% formaldehyde for 20 min at room temperature with gentle shaking. Experiments were performed with biological triplicates. Chromatin preparation was performed as described previously (57). Immunoprecipitation and library preparation for multiplex Illumina sequencing were carried out as described previously (58, 59). Illumina sequencing was performed on a HiSeq 2500 at the Gene Expression and Single Cell core facility of Faculty of Health Sciences at University of Macau. Sequencing data processing was carried out as described in reference 60. Bowtie2 (61) was used to align reads to the Aspergillus nidulans reference genome (version s10-m04-r03). CreA binding peaks were identified using MACS2 (62). ChIP-seq summits within 200 bp from different samples were merged as one by the mergePeaks program from the Homer software package (63). The reported ChIP-seq summits from mergePeaks were mapped to the closest gene to identify CreA target genes. CreA binding levels at target genes were calculated by counting normalized ChIP-seq read counts within a 200 bp window spanning the peak summit at corresponding promoters. For promoters with multiple CreA binding sites, the binding signal at each peak summit was calculated, and the sum for all peaks on the same promoter was reported. The violin plot was generated using FungiExpresZ (https://cparsania.shinyapps.io/FungiExpresZ/).

### Glucose transport.

Strains were grown from 10^7^ conidia in 30 ml complete medium for 24 h, washed twice with double-distilled water (ddH_2_O), and transferred to MM supplemented with 1% (wt/vol) glucose for 24 h. Two hundred microliters was collected from the culture supernatants at the designated time points, and residual glucose (percent residual glucose in the supernatant compared to that at time zero) was measured using the Glucose GOD-PAP Liquid Stable Mono-reagent kit (LaborLab Laboratories Ltd., Guarulhos, São Paulo, Brazil) according to manufacturer’s instructions.

### Intracellular glycogen and trehalose concentrations.

Intracellular glycogen and trehalose levels were measured in mycelia grown 24 h in MM supplemented with xylose as a carbon source as described previously (14).

### RNA extractions, cDNA biosynthesis, and qRT-PCRs.

RNA extractions of mycelia grown in biological triplicates were performed using TRIzol (Life Technologies), according to the manufacturer’s instructions. cDNA was synthesized from 1 μg RNA using the ImProm-II reverse transcriptase (Promega) kit, according to the manufacturer’s instructions. For each qRT-PCR, 1 μl cDNA was used, and the PCR set up was exactly as described previously (13). Gene expression was normalized by *tubC* (tubulin), whose expression remains constant under all tested conditions.

### Protein extraction and Western blotting.

Crude protein extracts from mycelia were obtained by extraction from ground mycelia with B250 buffer (250 mM NaCl, 100 mM Tris-HCl [pH 7.5], 10% glycerol, 1 mM EDTA, and 0.1% NP-40) supplemented with 1.5 ml/liter 1 M dithiothreitol (DTT), 1 pill/10 ml cOmplete Mini protease inhibitor cocktail, EDTA free (Roche), 3 ml/liter 0.5 M benzamidine, 1 pill/10 ml phosSTOP phosphatase inhibitors, and 10 ml/liter 100 mM phenylmethylsulfonyl fluoride (PMSF). Western blotting was carried out as described previously (24). Protein extracts from biological triplicates were used to carry out the Western blot experiments.

### Immunoprecipitation (GFP pulldown).

Total protein lysates from the WT (GFP-negative, negative control), the CreA-GFP (positive control), and the CreA-GFP phosphomutation-harboring strains grown in biological triplicates were centrifuged at 10,400 × *g* at 4°C for 10 min before the supernatant was collected into a new microcentrifuge tube. GFP-trap beads (Chromotek) were equilibrated with B250 lysis buffer (20 μl of beads into 500 μl lysis buffer B250) for 10 min before they were collected by centrifugation according to the manufacturer’s instructions and incubated with 6 mg of total protein lysate at 4°C for 3 h. After incubation with protein lysates, GFP-trap beads were collected, and the supernatant was removed. The GFP-trap beads were washed two times with 1 ml B250 lysis buffer without DTT, and one additional wash step was carried with the addition of DTT. GFP-trap beads were collected, and the supernatant was removed.

### Proteolytic digestion of protein after GFP pulldown.

Dried GFP-trap eluates were solubilized in 50 μl of 50 mM triethylammonium bicarbonate (TEAB) in 50:50 (vol/vol) trifluoroethanol (TFE) to water. Protein thiols were reduced and carbamidomethylated in one step (thermomixer at 70°C, 500 rpm, 30 min) by addition of 500 nmol tris(2-carboxyethyl)phosphine (TCEP) and 625 nmol 2-chloroacetamide (each solubilized in 100 mM aqueous TEAB). Subsequently, the protein solution was concentrated to a final volume of approximately 5 μl using a vacuum concentrator and diluted with 45 μl 100 mM TEAB to gain a final volume of 50 μl. Tryptic cleavages were performed using 1 μg rLys C (Promega number [no.] V1671) with incubation for 2 h at 37°C, and subsequently, 2 μg Trypsin Gold (Promega no. V5280) was added and the mixture was incubated for a further 16 h at 37°C. GluC cleavages were performed using 2 μg GluC (Promega no. V1651) and incubating for 18°C at 37°C. Peptides were dried and resolubilized in 25 μl 0.05% trifluoroacetic acid and 2% acetonitrile using a water bath sonicator (15 min). Finally, the samples were filtered through 0.2-μm spin filters (Merck Millipore Ultrafree-MC, hydrophilic polytetrafluoroethylene [PTFE] membrane, catalog no. UFC30LG25) at 18,000 × *g* for 15 min, and the filtrate was transferred into high-performance liquid chromatography (HPLC) vials.

### Phosphoproteome analysis by LC-MS/MS.

Proteome analysis, including phosphosite identification, was performed by nanoflow liquid chromatography coupled to tandem mass spectrometry (nLC-MS/MS) as recently published (64).

### Protein database search and data analysis.

Tandem mass spectra were searched against the FungiDB protein database of Aspergillus nidulans FGSC A4 (https://fungidb.org/common/downloads/release-44/AnidulansFGSCA4/fasta/data/FungiDB44_AnidulansFGSCA4_AnnotatedProteins.fasta), the protein sequence of CreA-GFP using Proteome Discoverer (PD) 2.2 (Thermo), and the algorithms of Mascot 2.4.1, Sequest HT (version of PD2.2), and MS Amanda 2.0. Two missed cleavages were allowed for both tryptic and GluC digestion. The precursor mass tolerance was set to 10 ppm, and the fragment mass tolerance was set to 0.02 Da. Modifications were defined as dynamic oxidation of Met, acetylation of the protein N terminus, phosphorylation of Ser, Thr, and Tyr, and either ubiquitination (GG, tryptic cleavage) or SUMOylation of Lys (QIGG/QIGGG, GluC cleavage) as well as static Cys carbamidomethylation. A strict false-discovery rate (FDR) of <1% was required for positive protein hits. The Percolator node of PD2.2 and a reverse decoy database was used for *q* value validation of spectral matches. Only rank 1 proteins and peptides of the top scored proteins were counted. The Minora algorithm of PD2.2 was applied for relative label-free quantification.

### Statistical analysis.

Statistical analysis was performed on a minimum of three biological replicates using a two-way analysis of variance (ANOVA) multiple-comparison test or a one-tailed *t* test (Prism GraphPad). Comparisons were carried out with reference to the wild-type strain under all conditions.

### Data availability.

The mass spectrometry proteomics data have been deposited to the ProteomeXchange Consortium via the PRIDE (65) partner repository with the data set identifier PXD018967. ChIP-seq results were submitted to NCBI under accession number SUB7547091.
